# Prognostic utility of body composition parameters based on computed tomography analysis of advanced non-small cell lung cancer treated with immune checkpoint inhibitors

**DOI:** 10.1186/s13244-023-01532-4

**Published:** 2023-10-26

**Authors:** Ji Eun Park, Jaemin Jo, Jeonghwan Youk, Miso Kim, Soon Ho Yoon, Bhumsuk Keam, Tae Min Kim, Dong-Wan Kim

**Affiliations:** 1 Department of Internal Medicine, Jeju National University Hospital, Jeju, South Korea; 2 Cancer Research Institute, Seoul National University, Seoul, South Korea; 3 Department of Internal Medicine, Seoul National University College of Medicine and Seoul National University Hospital, 101 Daehak-ro, Jongno-gu, Seoul, 03080 South Korea; 4 Department of Radiology, Seoul National University College of Medicine and Seoul National University Hospital, 101 Daehak-ro, Jongno-gu, Seoul, 03080 South Korea

**Keywords:** Non-small cell lung cancer, Immune checkpoint inhibitor, Visceral fat, Computed tomography, Body composition

## Abstract

**Objective:**

The purpose of this study was to evaluate the prognostic impact of body composition parameters based on computed tomography (CT) in patients with non-small cell lung cancer (NSCLC) who received ICI treatment.

**Methods:**

This retrospective study analyzed the data from advanced NSCLC patients treated with ICI therapy between 2013 and 2019. We included patients with NSCLC who underwent baseline CT scans. The exclusion criteria included patients who received three or more lines of chemotherapy, those with insufficient clinical information, or those without treatment response evaluation.

**Results:**

A total of 136 patients were enrolled. Among the volumetric body composition parameters, patients in the highest quartiles (Q2–4) of the visceral fat index (VFI) exhibited a higher response rate to ICI therapy than those in the lowest quartile (Q1) of VFI (Q1 vs. Q2–4: 18.2% vs. 43.1%, *p* = 0.012). Patients with a VFI in Q2–4 had significantly prolonged progression-free survival (PFS) and overall survival (OS) (PFS, Q1 vs. Q2–4: 3.0 months vs. 6.4 months, *p* = 0.043; OS, Q1 vs. Q2–4: 5.6 months vs. 16.3 months, *p* = 0.004). Kaplan–Meier analysis based on the VFI and visceral fat Hounsfield unit (HU) revealed that patients with VFI in Q1 and HU in Q2–4 had the worst prognosis.

**Conclusions:**

Visceral fat volume is significantly associated with treatment outcomes in ICI-treated patients with NSCLC. Moreover, fat quality may impact the treatment outcomes. This finding underscores the potential significance of both fat compartments and fat quality as prognostic indicators.

**Critical relevance statement:**

Visceral fat volume is significantly associated with treatment outcomes in ICI-treated patients with non-small cell lung cancer. Moreover, fat quality may impact the treatment outcomes. This finding underscores the potential significance of both fat compartments and fat quality as prognostic indicators.

**Graphical Abstract:**

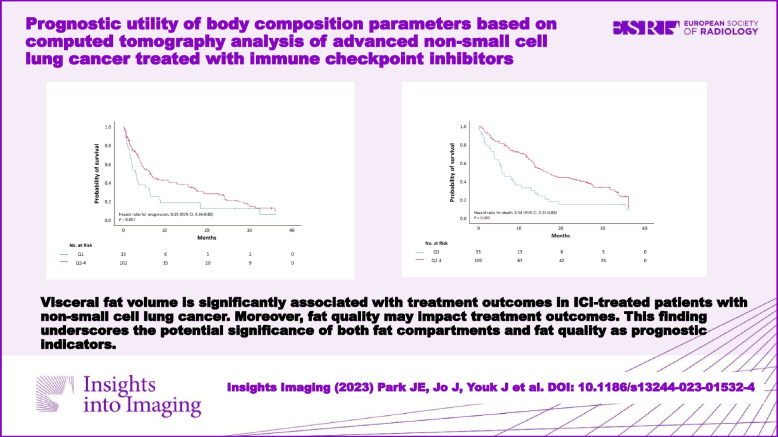

**Key points:**

• We found that visceral fat volume positively correlated with treatment response and survival in patients with non-small cell lung cancer receiving immune checkpoint inhibitors.

• Additionally, a trend toward a negative correlation between visceral fat attenuation and survival was observed.

• The findings highlight the prognostic utility of fat compartments and fat quality.

**Supplementary Information:**

The online version contains supplementary material available at 10.1186/s13244-023-01532-4.

## Introduction

Lung cancer is the leading cause of cancer-related mortality, with an estimated 2.2 million new cases recorded in 2020 [1]. Immune checkpoint inhibitors (ICIs) targeting programmed death-1 (PD-1) and programmed death ligand-1 (PD-L1) can improve survival outcomes in patients with advanced non-small cell lung cancer (NSCLC) and have become the standard treatment [2, 3]; however, their efficacy varies widely among patients, with some exhibiting primary or acquired resistance to ICIs and having poor prognoses [4–6]. The ability to identify patients likely to benefit from ICI therapy can lead to more personalized treatment plans, reduced side effects from unnecessary treatments, and cost savings. Moreover, for patients predicted to have low responsiveness to ICIs, alternative treatment options such as targeted therapies, chemotherapy, or participation in clinical trials for novel agents could be considered, emphasizing the importance of monitoring treatment response and adjusting treatment plans as needed [7, 8]. Therefore, numerous studies have sought to identify predictive biomarkers that can determine which patients are likely to benefit from ICI therapy. [9–11]. Established predictive biomarkers for ICIs, including PD-L1 expression, tumor mutation burden, microsatellite instability, and tumor-infiltrating lymphocytes, are mainly related to cancer itself or the associated tumor microenvironment [9, 12]. Nonetheless, studies have examined the predictive utility of host-related factors, such as sex, age, and obesity, to establish the efficacy of ICIs [13–15].

Obesity-related inflammation can dysregulate the immune response, substantially impacting the efficacy and toxicity of immunotherapy across several cancer types [16–20]. A previous preclinical study has revealed that obesity contributes to PD-1-mediated T cell dysfunction; however, it may increase the responsiveness of tumor cells to ICIs [21]. Moreover, growing evidence has suggested an association between obesity and improved outcomes in patients with NSCLC undergoing ICI therapy [22, 23]. However, studies that employed body mass index (BMI) as a surrogate for obesity have reported inconsistent results regarding the relationship between BMI and the efficacy of ICIs [6, 23–25].

As an independent factor, BMI may be insufficient to accurately represent the complexity and heterogeneity of body composition owing to its low sensitivity, as indicated by discrepancies between BMI and central obesity [26, 27]. Accordingly, studies have explored the relationships of other adipose tissue indicators with clinical outcomes in patients with cancer [23, 28]. For example, computed tomography (CT) allows volumetric measurement of body composition, which enables analysis using fat volume and quality instead of BMI-based obesity. The recent advancements in artificial and machine learning have enhanced the speed and accuracy of extracting body composition indicators from CT scans, and measurements of these parameters before treatment have been demonstrated to play a crucial role in predicting health, offering the potential to improve patient outcomes when applied clinically [29, 30].

Herein, we aimed to evaluate the relationship between body composition parameters, as determined through pre-treatment CT, and clinical outcomes in patients with NSCLC receiving ICI therapy.

## Methods

### Study design and patients

This study is a single-center, retrospective investigation conducted in accordance with the Declaration of Helsinki and approved by the institutional review board (IRB No. 2001-069-1094). Informed consent was waived as the study relied on anonymous clinical data and images. We reviewed the electronic medical records of patients with advanced NSCLC who received ICI therapy between 2013 and 2019. The inclusion criteria were as follows: (a) patients with pathologically confirmed NSCLC aged 18 years or older, (b) patients who underwent baseline chest and abdominopelvic CT scans, and (c) patients who received ICI therapy. We excluded the following cases: (a) cases where ICI was administered after three or more lines of chemotherapy, (b) patients who had received prior ICI therapy, (c) patients with other malignancies requiring treatment, (d) patients without a baseline CT scan, (e) patients with insufficient clinical information, and (f) patients who did not undergo treatment response evaluation during ICI therapy.

### Clinicopathological parameters and treatment outcomes

We collected data on the patient’s age at the time of ICI treatment initiation, sex, BMI (kg/m^2^), European Cooperative Oncology Group performance status (ECOG PS), smoking status, histology, driver oncogene mutations, PD-L1 immunohistochemistry findings, previous systemic therapies, treatment outcomes, and survival status. Furthermore, the treatment response and progression-free survival (PFS) were evaluated using Response Evaluation Criteria in Solid Tumors, version 1.1.

### CT protocol

All contrast-enhanced CT scans were obtained during the portal venous phase using one of the following multi-detector CT scanners from four manufacturers: Philips Medical Systems (Ingenuity CT [*n* = 31], Brilliance 64 [*n* = 16], iCT 256 [*n* = 5]), GE Healthcare (Discovery CT750 HD [*n* = 28], Revolution [*n* = 21]; Siemens Healthineers (SOMATOM Definition [*n* = 17], SOMATOM Force [*n* = 4]), and Canon Medical Systems (Aquilion One [*n* = 14]). The scans were acquired using voltage settings of 100 to 140 kVp (100 kVp [*n* = 53], 120 kVp [*n* = 74], and 140 kVp [*n* = 9]) with automatic exposure control. All CT images were reconstructed with a soft tissue kernel and had a slice thickness of 3 mm or less.

### CT analysis

Abdominal CT images were imported into a commercially available deep learning-based body composition analysis software (DeepCatch, v1.0.0.0, MEDICALIP Co. Ltd., Seoul, Korea). The software comprised a three-dimensional (3D) U-Net that segmented CT images into seven classes: skin, muscle, bone, abdominal visceral fat, subcutaneous fat, internal organs/vessels, and central nervous system. In the external validation, the 3D U-Net achieved an average Dice score of 92.3–99.3% for muscle, visceral fat, and subcutaneous fat [31]. A representative CT image is shown in Additional file 1: Fig. S1. An experienced radiologist (SHY) with 17 years of experience in body CT interpretation reviewed the segmentation results obtained with the software and revised the results as appropriate. Subsequently, the software yielded CT-derived parameters, including total fat volume (cm^3^), visceral fat volume (cm^3^), subcutaneous fat volume (cm^3^), skeletal muscle volume (cm^3^), visceral fat attenuation (Hounsfield units [HUs]), and subcutaneous fat attenuation (HU) at the waist, which was defined according to the World Health Organization definition as “between the 12th rib and the iliac crest” [32]. The total fat volume, visceral fat volume, subcutaneous fat volume, and skeletal muscle area were normalized for height in square meters to calculate the total fat index (TFI), visceral fat index (VFI), subcutaneous fat index (SFI), and skeletal muscle index (SMI), respectively. Given the lack of established cutoff values for fat volume indexes and fat attenuation for evaluating survival outcomes, we arbitrarily split the data into the lowest (Q1) and highest (Q2–4) quartiles. The Q1 cutoff values for TFI, VFI, and SFI were 404.1, 147.6, and 235.0, respectively. The SMI cutoff value was based on the Q1 for each sex (297.0 for males and 257.4 for females)

### Statistical analysis

Categorical variables were compared using Pearson’s chi-square test. Univariate and multivariate logistic regression analyses were performed to evaluate the relationships between variables and the objective response to ICI therapy. PFS was defined as the period from initiating ICI treatment to clinical or radiographic progression or death. The overall survival (OS) was defined as the period from initiating ICI treatment to the date of the last follow-up or death. The follow-up period was set at 36 months. The median PFS and OS were calculated using the Kaplan–Meier method. Survival outcomes were compared using the log-rank test. Multivariate analysis of prognostic factors for survival was performed using the Cox proportional hazard model with a forward stepwise method. All statistical tests were two-sided, with statistical significance set at *p* < 0.05. All statistical analyses were performed using SPSS^®^ Statistics, version 22.0 (IBM Corp., Armonk, NY, USA).

## Results

### Patient characteristics and treatment response

Out of 228 advanced NSCLC patients treated with ICI, 92 were excluded based on the exclusion criteria, leaving a total of 136 patients enrolled in this study (Additional file 1: Fig. S2). Table 1 summarizes their baseline characteristics. The relationship between clinicopathological variables and the response to ICI treatment is shown in Table 2. Factors related to the treatment response included age, sex, and the number of lines of prior systemic therapy.

**Table 1:** Baseline characteristics of the patients

Characteristics	Patients (*n* = 135), no. (%)
Age, years, median (range)	66 (37–93)
Sex	
Female	30 (22.2)
Male	105 (77.8)
BMI status, kg/m^2^, median (range)	23.0 (15–31)
Under-weight (< 18.5 kg/m^2^)	14 (10.4)
Normal (18.5 ≤ BMI < 23 kg/m^2^)	53 (39.3)
Over-weight (23 ≤ BM I < 25 kg/m^2^)	37 (27.4)
Obese (≥ 25 kg/m^2^)	31 (23.0)
Smoking status	
Never smoker	37 (27.4)
Ever-smoker	98 (72.6)
ECOG PS	
0–1	129 (95.6)
≥ 2	6 (4.4)
Histologic type	
Adenocarcinoma	67 (49.6)
Squamous cell carcinoma	41 (30.4)
Others^a^	27 (20.0)
PD-L1 expression	
Negative	28 (20.7)
Positive	72 (53.3)
Unknown	35 (25.9)
Type of ICI	
Anti-PD-1 or anti-PD-L 1 monotherapy	105 (77.8)
ICI-based combination therapy	30 (22.2)
No. of lines of prior chemotherapy	
0	30 (22.2)
1	70 (51.9)
2	35 (25.9)
Cause of ICI discontinuation	
Progression	98 (72.6)
Toxicity	12 (8.9)
Others	1 (0.7)
Best response to ICI	
Partial response	50 (37.0)
Stable disease	37 (27.4)
Progressive disease	48 (35.6)

*BMI*, body mass index; *ECOG PS*, Eastern Cooperative Oncology Group performance status; *ICI*, immune checkpoint inhibitor; *PD-1*, programmed death-1; *PD-L1*, programmed death ligand-1

^a^Others included sarcomatoid, mucinous, and poorly differentiated carcinoma

**Table 2:** Correlations between body composition parameters and clinicopathological variable with tumor response to immune checkpoint inhibitors

Variables		Tumor response		*p v*alue
		PR	SD/PD	
Age	< 65	13 (24.1%)	41 (75.9%)	0.012
	≥ 65	37 (45.7%)	44 (54.3%)	
Sex	Female	45 (42.9%)	60 (57.1%)	0.010
	Male	5 (16.7%)	25 (83.3%)	
BMI	< 25	35 (33.0%)	71 (67.0%)	0.083
	≥ 25	15 (51.7%)	14 (48.3%)	
Smoking status	Never smoker	9 (24.3%)	28 (75.7%)	0.075
	Ever-smoker	41 (41.8%)	57 (58.2%)	
ECOG PS	0–1	49 (38.0%)	80 (62.0%)	0.412
	≥ 2	1 (16.7%)	5 (83.3%)	
Histologic type	Squamous cell carcinoma	11 (26.8%)	30 (73.2%)	0.123
	Non-squamous cell carcinoma	39 (41.5%)	55 (58.5%)	
PD-L1 expression	Negative	7 (25.0%)	21 (75.0%)	0.097
	Positive	31 (43.1%)	41 (56.9%)	
Type of ICI	Anti-PD-1 or anti-PD-L1 monotherapy	39 (37.1%)	66 (63.3%)	1.000
	ICI-based combination therapy	11 (36.7%)	19 (63.3%)	
No. of lines of prior chemotherapy	0	17 (56.7%)	13 (43.3%)	0.018
	≥ 1	33 (31.4%)	72 (68.6%)	
Skeletal muscle index	Q1	12 (37.5%)	20 (62.5%)	0.951
	Q2–4	38 (36.9%)	65 (63.1%)	
Total fat index	Q1	10 (29.4%)	24 (70.6%)	0.313
	Q2–4	40 (39.6%)	61 (60.4%)	
Visceral fat index	Q1	6 (18.2%)	27 (81.8%)	0.012
	Q2–4	44 (43.1%)	58 (56.9%)	
Subcutaneous fat index	Q1	12 (35.3%)	22 (64.7%)	0.898
	Q2–4	38 (37.6%)	63 (62.4%)	

*BMI*, body mass index; *ECOG PS*, Eastern Cooperative Oncology Group performance status; *ICI*, immune checkpoint inhibitor; *PD-1*, programmed death-1; *PD-L1*, programmed death ligand-1; *PD*, progressive disease; *PR*, partial response; *Q1*, lowest quartile; *Q2–4*, highest quartiles; *SD*, stable disease

### Volumetric parameters and differences in treatment outcomes by volumetric parameters

The median SMI, TFI, VFI, and SFI values were 333.6 (range, 45.1–533.0 cm^3^/m^2^), 598.0 (range, 30.8–1467.7 cm^3^/m^2^), 264.0 (range, 6.4–821.8 cm^3^/m^2^), and 314.4 (range, 24.3–764.9 cm^3^/m^2^), respectively. Fewer patients had a high VFI in the stable disease/progressive disease group (58/85, 68.2%) than in the partial response group (44/50, 88%) (*p* = 0.012, Table 2), with the logistic regression analysis showing a similar trend (Additional file 1: Table S1). Significantly higher proportions of patients had initial progressive disease as best response on ICIs in the Q1 group of TFI (Q1 vs. Q2–4: 50.0 % vs. 30.7%, *p* = 0.042) and Q1 group of VFI (Q1 vs. Q2–4: 60.6% vs. 27.5%, *p* = 0.001, data not shown) compared with the corresponding Q2–4 groups. There were no differences in SMI and SFI between the groups with different treatment responses.

### Differences in survival outcomes by volumetric parameters

**Fig. 1:**
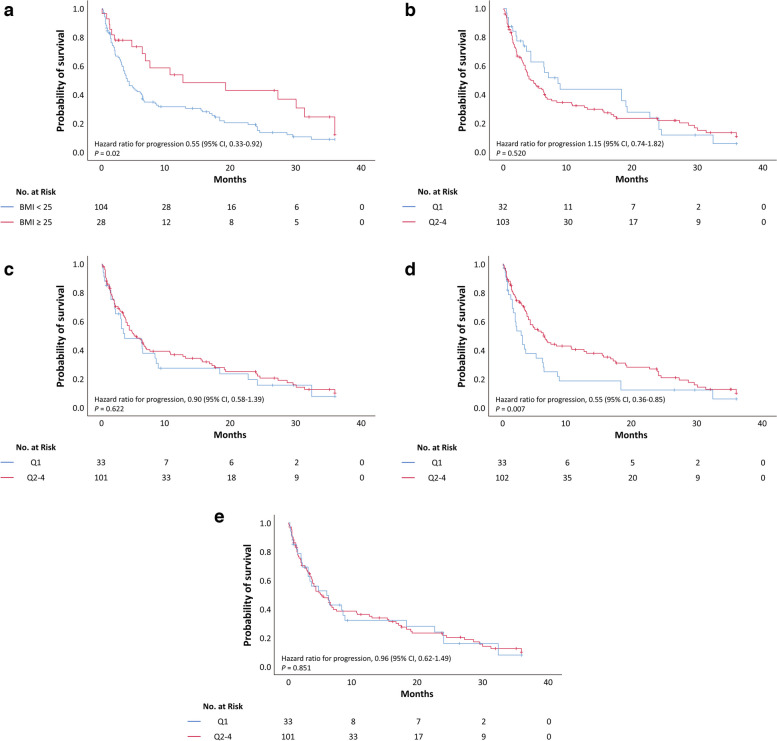
Kaplan–Meier survival curves for progression-free survival according to body composition parameters. **a** Body mass index. **b** Skeletal muscle index. **c** Total fat index. **d** Visceral fat index. **e** Subcutaneous fat index. The cutoff of each parameter is the lowest quartile (Q1)

**Fig. 2:**
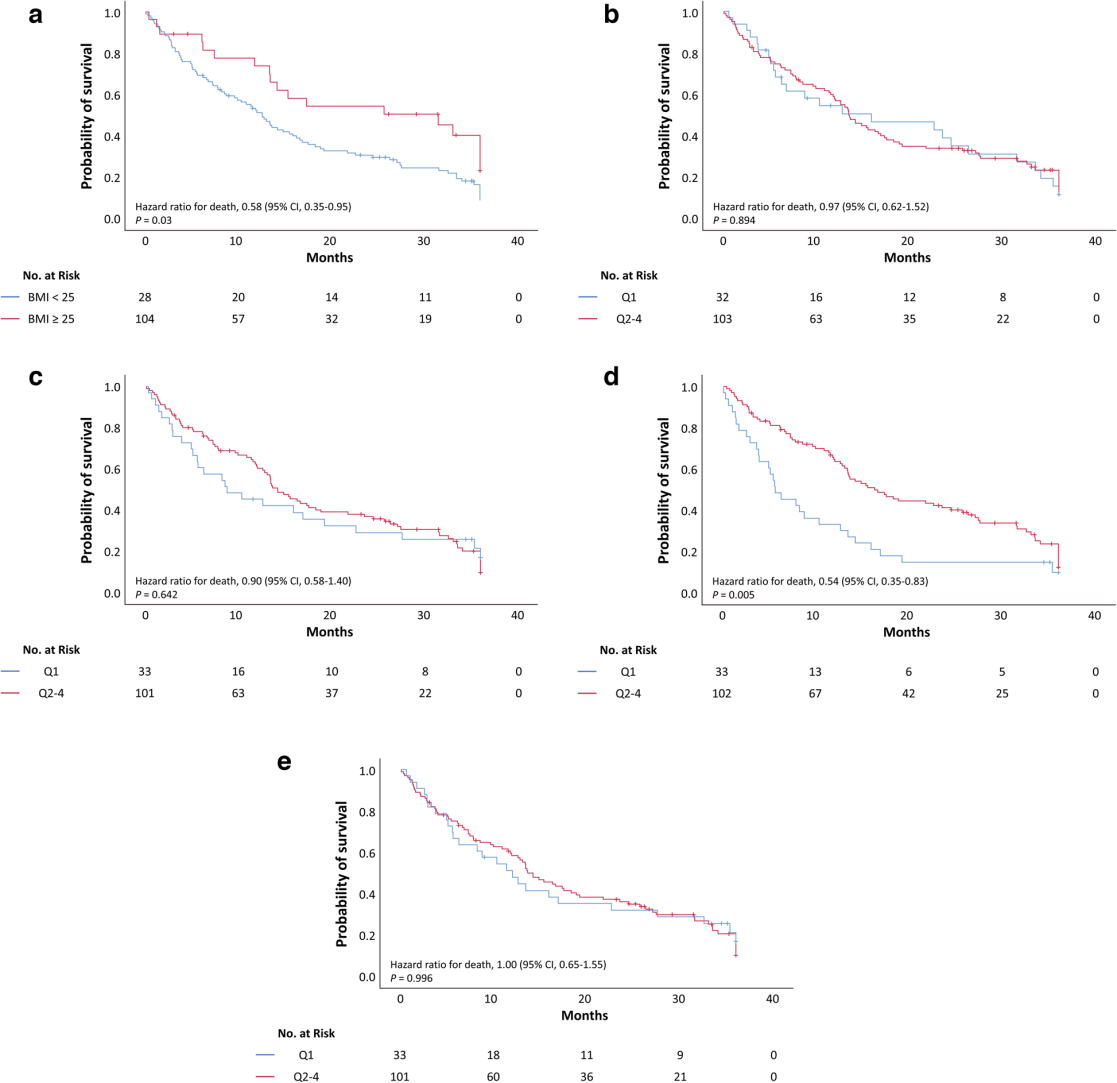
Kaplan–Meier survival curves for overall survival according to body composition parameters. **a** Body mass index. **b** Skeletal muscle index. **c** Total fat index. **d** Visceral fat index. **e** Subcutaneous fat index. The cutoff for each parameter is the lowest quartile (Q1)

Kaplan–Meier survival analysis was performed using BMI and volumetric parameters. Obesity was defined as a BMI of 25 kg/m^2^ or higher [33]. Patients with obesity had a significantly longer PFS than those without obesity (BMI < 25 vs. ≥ 25 kg/m^2^; 3.9 months vs. 12.5 months, *p* = 0.025 using the log-rank test) (Fig. 1). Moreover, the Q2–4 group of VFI had a significantly longer PFS than the Q1 group (Q1 vs. Q2–4: 3.0 months vs. 6.4 months, *p* = 0.043 by log-rank test). Similar findings were obtained for the median OS in the obese group (BMI < 25 vs. ≥ 25 kg/m^2^: 12.6 months vs. 31.5 months, *p* = 0.024 by log-rank test) and Q2–4 group of VFI (Q1 vs. Q2–4: 5.6 vs. 16.3 months, *p* = 0.004 by log-rank test) (Fig. 2). However, no significant between-group differences were observed for other parameters, including SMI, TFI, and SFI.

We performed a Cox proportional hazard analysis to assess the impact of the clinicopathological and volumetric parameters on PFS and OS. In the multivariate analysis, VFI was an independent indicator for improved PFS (Q1 vs. Q2–4: hazard ratio [HR] 0.497, *p* = 0.004 using Cox proportional hazards regression) and OS (Q1 vs. Q2–4: HR 0.466, *p* = 0.002 using Cox proportional hazards regression) (Table 3).

**Table 3:** Cox proportional hazard analysis of the prognostic factors for progression-free survival and overall survival

**Variable**	**Progression-free survival**						**Overall survival**					
	**Univariable analysis**			**Multivariable analysis**			**Univariable analysis**			**Multivariable analysis**		
	**HR**	**95% CI**	***p* value**	**HR**	**95% CI**	***p* value**	**HR**	**95% CI**	***p* value**	**HR**	**95% CI**	***p* value**
**Age, years**			0.103						0.225			
< 65	1						1					
≥ 65	0.723	0.489–1.068					0.788	0.536–1.158				
**Sex**			0.009						0.047			
Female	1						1					
Male	1.796	1.161–2.779					1.550	1.006–2.380				
**BMI, kg/m**^**2**^			0.021						0.030			
< 25	1						1					
≥ 25	0.550	0.330–0.915					0.576	0.350–0.949				
**ECOG PS**			0.047			0.051			0.017			0.013
0	1			1			1			1		
≥ 1	2.325	1.013–5.338		2.319	0.996–5.400		2.766	1.197–6.390		2.934	1.253–6.867	
**Smoking status**			0.027			0.011			0.069			0.021
Never smoker	1			1			1			1		
Ever-smoker	0.626	0.414–0.949		0.567	0.367–0.877		0.674	0.446–1.018		0.603	0.392–0.926	
**Histologic type**			0.178			0.022			0.112			0.013
Squamous cell carcinoma	1			1			1			1		
Non-squamous cell carcinoma	0.755	0.501–1.137		0.588	0.373–0.925		0.738	0.492–1.108		0.568	0.363–0.890	
**PD-L1 expression**			0.855						0.478			
Negative	1						1					
Positive	1.048	0.635–1.727					0.835	0.507–1.374				
**Type of ICI**			0.054						0.025			
Anti-PD-1 or anti-PD-L1 monotherapy	1						1					
ICI-based combination therapy	0.613	0.372–1.009					0.566	0.344–0.930				
**No. of lines of prior systemic therapy**			0.001			0.001			0.001			0.002
0	1			1			1			1		
≥ 1	2.388	1.416–4.028		2.368	1.396–4.018		2.275	1.367–3.788		2.266	1.357–3.785	
**Skeletal muscle index**			0.520						0.894			
Q1	1						1					
Q2-4	1.160	0.738–1.821					0.970	0.619–1.520				
**Total fat index**			0.622						0.642			
Q1	1						1					
Q2–4	0.895	0.577–1.390					0.901	0.581–1.397				
**Visceral fat index**			0.007			0.004			0.005			0.002
Q1	1			1			1			1		
Q2–4	0.554	0.360–0.853		0.497	0.307–0.804		0.542	0.353–0.834		0.466	0.290–0.748	
**Subcutaneous fat index**			0.851						0.996			
Q1	1						1					
Q2–4	0.959	0.618–1.488					0.999	0.645–1.548				

*CI*, confidence interval; *HR*, hazard ratio; *BMI*, body mass index; *ECOG PS*, Eastern Cooperative Oncology Group performance status; *ICI*, immune checkpoint inhibitor; *PD-1*, programmed death-1; *PD-L1*, programmed death ligand-1; *Q1*, lowest quartile; *Q2–4*, highest quartiles

**Fig. 3:**
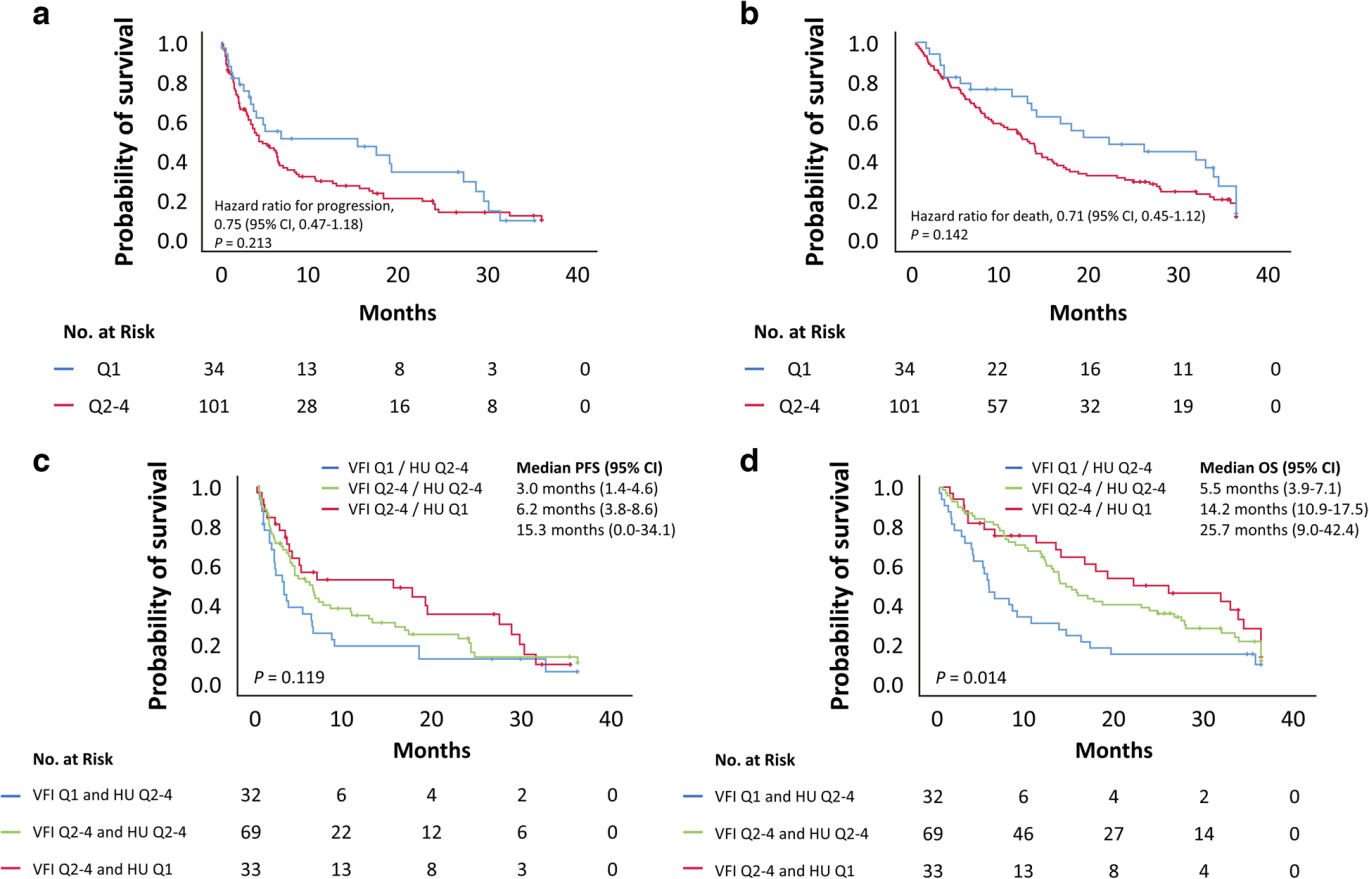
Kaplan–Meier survival curves. **a** Progression-free survival. **b** Overall survival according to visceral fat Hounsfield unit. **c** Progression-free survival. **d** Overall survival according to visceral fat index and visceral fat Hounsfield unit

Finally, to investigate not only the volume of fat but also the impact of fat quality on treatment outcomes, we performed a Kaplan–Meier survival analysis using a visceral fat HU cutoff value of − 91.13 (the first quartile of median HU). As shown in Fig. 3, there was a trend toward a shorter PFS and OS in the Q2–4 group of visceral fat HU than in the Q1 group (median PFS, Q1 vs. Q2–4: 15.3 months vs. 4.2 months, *p* = 0.213 using the log-rank test; median OS, Q1 vs. Q2–4: 21.7 months vs. 12.8 months, *p* = 0.142 using the log-rank test, Fig. 3). Subsequently, the study cohort was divided into four groups based on the VFI and visceral fat HU cutoff values; however, only one patient had both VFI and visceral fat HU in Q1 and was excluded from the analysis. Patients in the Q1 group of VFI who were also in the Q2–4 group of visceral fat HU had the shortest PFS and OS (median PFS = 3.0 months, 95% confidence interval [1.4–4.6 months]; median OS = 5.5 months, 95% confidence interval [3.8–7.2 months]; Fig. 3).

## Discussion

In the present study, we examined the predictive utility of CT-based body composition parameters for treatment response and prognosis in patients with NSCLC who received ICI therapy. We found that the visceral fat volume positively correlated with the treatment response and survival.

Obesity is a well-established cause of cancer [34]. Specifically, a high BMI is a risk factor for the development of and mortality from several cancer types, including breast, colorectal, and kidney cancer [35]. Conversely, a high BMI has been shown to improve survival in patients with other types of cancers, and this phenomenon has been termed the “obesity paradox” [36]. Obesity can influence treatment response and related toxicities [37, 38]. Recent studies have investigated the role of obesity in patients with cancer undergoing treatment with ICIs [39], with some studies reporting improved treatment responses to ICIs in patients with obesity [16] and others demonstrating that obesity only improves PFS but not OS [40]. Nonetheless, most studies have demonstrated that treatment outcomes for ICI appear to be improved in patients with cancer with a high BMI [41–43].

Obesity contributes to chronic inflammatory conditions via multifaceted mechanisms. Specifically, obesity is associated with increased levels of leptin, free fatty acids, and pro-inflammatory cytokines. Additionally, obesity upregulates PD-1 expression in T cells and myeloid-derived suppressive cells. Collectively, the anti-tumor effects of ICIs could be amplified in the obesity-mediated inflammatory environment [39, 44].

It should be noted that BMI is not an optimal surrogate for obesity, given that it fails to accurately reflect body composition [28, 45–47]. Accordingly, studies have attempted to establish body composition parameters that better reflect obesity. Recent studies have explored the correlation between treatment outcomes and body composition parameters obtained through CT or positron emission tomography CT in patients with NSCLC treated with ICIs [23, 24]. One study, for example, explored the association between measures of skeletal muscle mass and adiposity (i.e., intramuscular, visceral, and subcutaneous adipose tissue) and changes during treatment, with a focus on disease progression and OS in patients with advanced lung cancer receiving immunotherapy [48]. The evaluation of the response to ICI treatment by measuring CT-based body composition at baseline, as done in the present study, is similar to this previous research. However, a key difference is that the correlation with disease progression was analyzed by calculating the delta value after early CT evaluation within 1–2 months, which allowed for the prediction of response changes [49]. In the present study, we evaluated the body composition using CT-based 3D volumetric analysis, which allows precise qualitative and quantitative analysis of adiposity. Visceral fat volume was associated with improved response and prolonged survival in patients with NSCLC who received ICI therapy. Additionally, visceral fat attenuation showed a tendency of a negative correlation with OS. Furthermore, patients with low VFI and high attenuation had the worst survival.

Visceral and subcutaneous fat display distinct anatomical distributions and gene expression profiles. Studies have reported differences in the expression of inflammation-related genes between abdominal visceral fat and subcutaneous fat [50, 51]. According to a previous study, pro-inflammatory cytokine genes were more abundant in the subcutaneous fat than in the visceral fat [51]. Furthermore, a previous study has reported a positive correlation between cancer mortality and fat attenuation [52]. Fat attenuation may be positively correlated with adipose tissue fibrosis, which is closely related to inflammation and cytokine release. Therefore, the fat compartment and quality may be more important than fat volume in immune regulation and response to ICI in patients with cancer [34].

In contrast to a previous report [53], we observed no correlation between SMI and clinical outcomes. Given that previous studies have used inconsistent criteria for sarcopenia, the optimal cutoff values for sarcopenia according to race, sex, or cancer type are yet to be established. A limitation of the present study is that it was a relatively small-scale retrospective study. In addition, the automatic CT segmentation method in this study does necessitate expert verification to confirm accurate segmentation, even though the deep learning algorithm showed high accuracy compared to the reference during its development [31]. However, this method greatly diminishes both time and computational demands, enabling more efficient analysis of extensive CT datasets. Additionally, we did not compare the CT-based body composition parameters with the results of other modalities, including dual-energy X-ray absorptiometry or bioelectrical impedance analysis; however, CT is considered the reference standard for assessing body composition [54]. Nonetheless, this study presents robust findings indicating the potential prognostic utility of abdominal visceral fat volume and attenuation in patients undergoing immunotherapy. A personalized management strategy for these patients could be developed by incorporating body composition parameter assessments into risk stratification and implementing targeted nutritional interventions.

## Conclusions

We observed a significant association between visceral fat volume and treatment outcomes in patients with NSCLC who received ICI therapy, indicating the prognostic utility of fat compartments and fat quality. Future large-scale prospective studies are warranted to confirm our findings.

### Supplementary Information

**Additional file 1:** **Table S1.** Logistic regression hazard analysis of the prognostic factors for tumor response. CI, confidence interval; HR, hazard ratio; BMI, body mass index; ECOG PS, Eastern Cooperative Oncology Group performance status; ICI, immune checkpoint inhibitor; OR, odds ratio; PD-L1, programmed death ligand-1. **Fig. S1.** Representative image of CT scan analysis. The image in the upper left corner shows 3D, axial, sagittal, and coronal views (clockwise). In the 3D view, there are three transverse planes, and the two green planes represent the waist range (lowest rib to iliac crest), while the middle light green plane indicates the L3 level. **Fig. S2.** CONSORT Diagram.

## Data Availability

The datasets generated or analyzed during the study are available from the corresponding authors upon reasonable request.

## Abbreviations

3D: Three-dimensional
BMI: Body mass index
CT: Computed tomography
ECOG PS: European Cooperative Oncology Group performance status
HR: Hazard ratio
HU: Hounsfield unit
ICI: Immune checkpoint inhibitor
NSCLC: Non-small cell lung cancer
OS: Overall survival
PD-1: Programmed death-1
PD-L1: Programmed death ligand-1
PFS: Progression-free survival
Q1: Lowest quartile
Q2–4: Highest quartiles
SFI: Subcutaneous fat index
SMI: Skeletal muscle index
TFI: Total fat index
VFI: Visceral fat index
