# Supplementary material for: Prognostic utility of body composition parameters based on computed tomography analysis of advanced non-small cell lung cancer treated with immune checkpoint inhibitors
Source: Insights Imaging. 2023 Oct 26;14:182. doi: 10.1186/s13244-023-01532-4 (PMC10600077; doi:10.1186/s13244-023-01532-4)
Supplement: Supplementary file 1 — Additional file 1: Table S1. Logistic regression hazard analysis of the prognostic factors for tumor response. CI, confidence interval; HR, hazard ratio; BMI, body mass index; ECOG PS, Eastern Cooperative Oncology Group performance status; ICI, immune checkpoint inhibitor; OR, odds ratio; PD-L1, programmed death ligand-1. Fig. S1. Representative image of CT scan analysis. The image in the upper left corner shows 3D, axial, sagittal, and coronal views (clockwise). In the 3D view, there are three transverse planes, and the two green planes represent the waist range (lowest rib to iliac crest), while the middle light green plane indicates the L3 level. Fig. S2. CONSORT Diagram. [file 13244_2023_1532_MOESM1_ESM.docx]

**Prognostic Utility of Body Composition Parameters based on Computed Tomography Analysis of Advanced Non-small Cell Lung Cancer Treated with Immune Checkpoint Inhibitors**

**ELECTRONIC SUPPLEMENTARY MATERIAL**

**Table S1:** Logistic regression hazard analysis of the prognostic factors for tumor response

| Variables | Univariable analysis | | |  | Multivariable analysis | | |
| --- | --- | --- | --- | --- | --- | --- | --- |
|  | OR | 95% CI | *P* value |  | OR | 95% CI | *P* value |
| Age, years |  |  | 0.012 |  |  |  | 0.019 |
| <65 | 1 |  |  |  | 1 |  |  |
| >65 | 0.377 | 0.176–0.808 |  |  | 0.365 | 0.157–0.849 |  |
| **Sex** |  |  | 0.013 |  |  |  | 0.030 |
| Female | 1 |  |  |  | 1 |  |  |
| Male | 3.750 | 1.322–10.557 |  |  | 3.543 | 1.131–11.100 |  |
| BMI, kg/m^2^ |  |  | 0.460 |  |  |  |  |
| <25 | 1 |  |  |  |  |  |  |
| >25 | 0.460 | 0.200–1.059 |  |  |  |  |  |
| ECOG PS |  |  | 0.313 |  |  |  |  |
| 0 | 1 |  |  |  |  |  |  |
| >1 | 3.062 | 0.347–26.991 |  |  |  |  |  |
| Smoking status |  |  | 0.069 |  |  |  |  |
| Never smoker | 1 |  |  |  |  |  |  |
| Ever smoker | 0.447 | 0.191–1.047 |  |  |  |  |  |
| Histologic type |  |  | 0.108 |  |  |  | 0.004 |
| Squamous cell carcinoma | 1 |  |  |  | 1 |  |  |
| Non-squamous cell carcinoma | 0.517 | 0.232–1.155 |  |  | 0.257 | 0.101–0.651 |  |
| PD-L1 expression |  |  | 0.209 |  |  |  |  |
| Negative | 1 |  |  |  |  |  |  |
| Positive | 0.713 | 0.421–1.209 |  |  |  |  |  |
| Type of ICI |  |  | 0.962 |  |  |  |  |
| Anti-PD 1 or Anti-PD-L 1 monotherapy | 1 |  |  |  |  |  |  |
| ICI-based combination therapy | 1.021 | 0.440–2.368 |  |  |  |  |  |
| No. of lines of prior systemic therapy |  |  | 0.013 |  |  |  | 0.047 |
| 0 | 1 |  |  |  | 1 |  |  |
| ≥1 | 2.853 | 1.242–6.552 |  |  | 2.546 | 1.013–6.399 |  |
| Skeletal muscle index |  |  | 0898 |  |  |  |  |
| Q1 | 1 |  |  |  |  |  |  |
| Q2–4 | 0.952 | 0.447–2.027 |  |  |  |  |  |
| Total fat index |  |  | 0.289 |  |  |  |  |
| Q1 | 1 |  |  |  |  |  |  |
| Q2–4 | 0.635 | 0.275–1.470 |  |  |  |  |  |
| Visceral fat index |  |  | 0.013 |  |  |  | 0.017 |
| Q1 | 1 |  |  |  | 1 |  |  |
| Q2–4 | 0.293 | 0.111–0.771 |  |  | 0.271 | 0.093–0.790 |  |
| Subcutaneous fat index |  |  | 0.808 |  |  |  |  |
| Q1 | 1 |  |  |  |  |  |  |
| Q2–4 | 0.904 | 0.402–2.034 |  |  |  |  |  |

CI, confidence interval; HR, hazard ratio; BMI, body mass index; ECOG PS, Eastern Cooperative Oncology Group performance status; ICI, immune checkpoint inhibitor; OR, odds ratio; PD-L1, programmed death ligand-1.


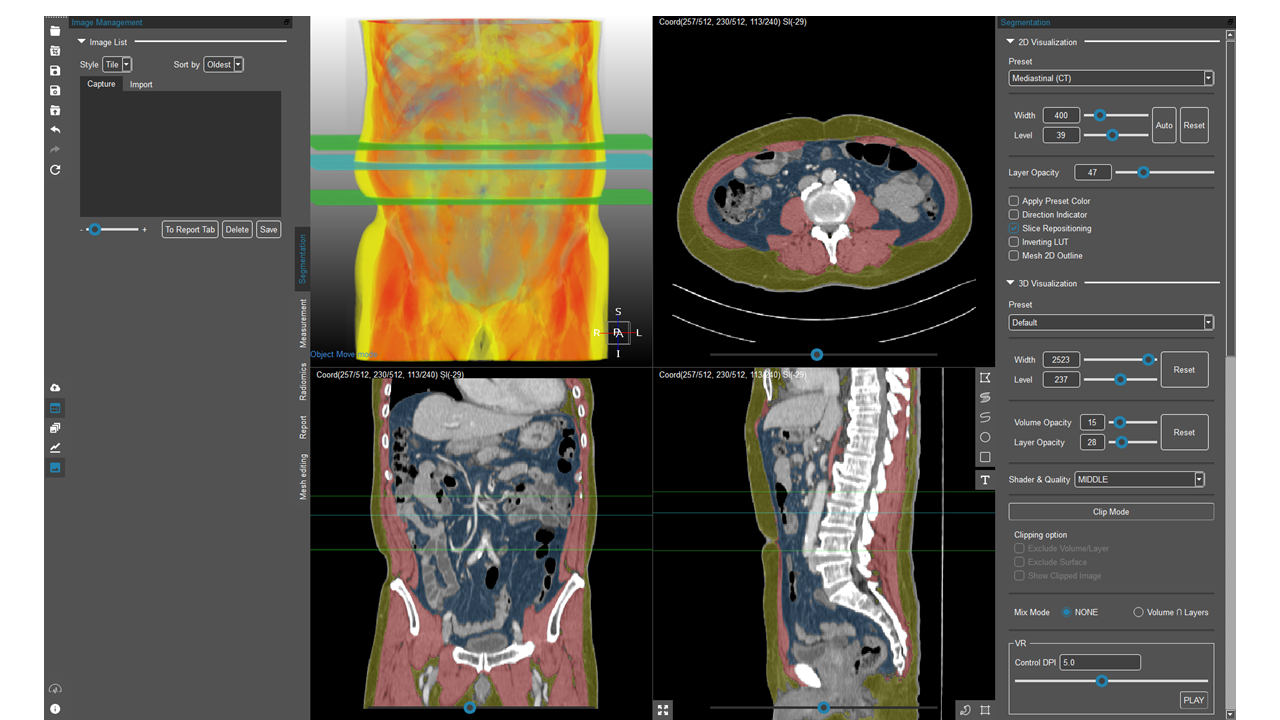


**Supplementary Figure S1:** Representative image of CT scan analysis.

The image in the upper left corner shows 3D, axial, sagittal, and coronal views (clockwise). In the 3D view, there are three transverse planes, and the two green planes represent the waist range (lowest rib to iliac crest), while the middle light green plane indicates the L3 level.


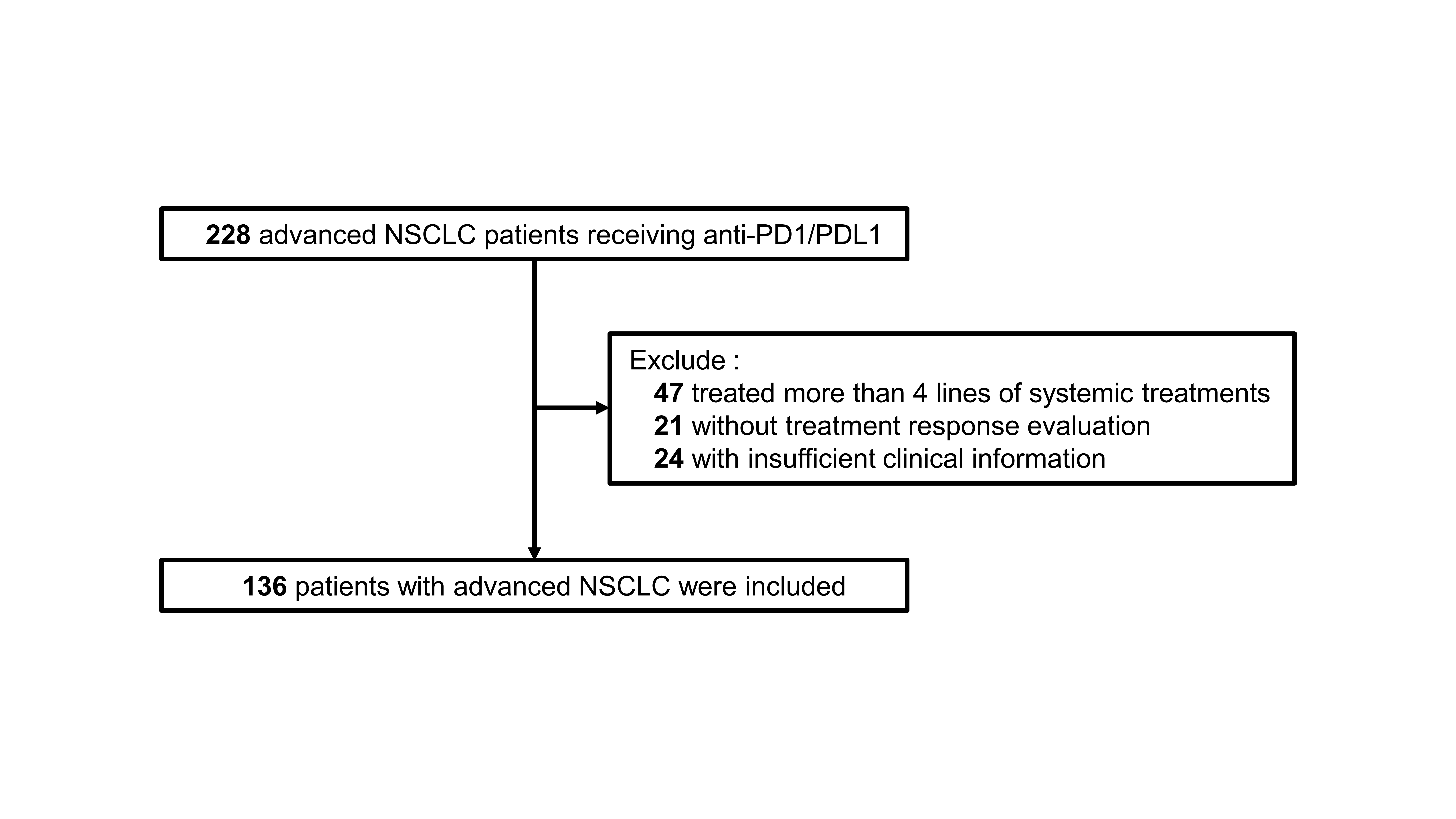


**Supplementary Figure S2:** CONSORT Diagram
